# Live imaging and analysis of postnatal mouse retinal development

**DOI:** 10.1186/1471-213X-13-24

**Published:** 2013-06-10

**Authors:** Philip EB Nickerson, Kara M Ronellenfitch, Nicklaus F Csuzdi, Jamie D Boyd, Perry L Howard, Kerry R Delaney, Robert L Chow

**Affiliations:** 1 Department of Biology, University of Victoria, Station CSC, PO Box 3020, Victoria, BC V8W 3N5, Canada; 2 FRAN, University of Guelph, Guelph, ON, Canada; 3 Department of Psychiatry, University of British Columbia, Vancouver, BC, Canada; 4 Department of Biochemistry/Microbiology, University of Victoria, Victoria, BC, Canada; 5 Current address: Department of Surgery (Neurosurgery), Dalhousie University, Halifax, NS B3H 3A7, Canada

**Keywords:** Postnatal retina, *in vitro* electroporation, *ex vivo* culture, Live 2-photon microscopy, Hierarchical cluster analysis

## Abstract

**Background:**

The explanted, developing rodent retina provides an efficient and accessible preparation for use in gene transfer and pharmacological experimentation. Many of the features of normal development are retained in the explanted retina, including retinal progenitor cell proliferation, heterochronic cell production, interkinetic nuclear migration, and connectivity. To date, live imaging in the developing retina has been reported in non-mammalian and mammalian whole-mount samples. An integrated approach to rodent retinal culture/transfection, live imaging, cell tracking, and analysis in structurally intact explants greatly improves our ability to assess the kinetics of cell production.

**Results:**

In this report, we describe the assembly and maintenance of an *in vitro*, CO_2_-independent, live mouse retinal preparation that is accessible by both upright and inverted, 2-photon or confocal microscopes. The optics of this preparation permit high-quality and multi-channel imaging of retinal cells expressing fluorescent reporters for up to 48h. Tracking of interkinetic nuclear migration within individual cells, and changes in retinal progenitor cell morphology are described. Follow-up, hierarchical cluster screening revealed that several different dependent variable measures can be used to identify and group movement kinetics in experimental and control samples.

**Conclusions:**

Collectively, these methods provide a robust approach to assay multiple features of rodent retinal development using live imaging.

## Background

The past decade has witnessed the evolution of *ex-vivo* retinal procurement and culture methodologies to study the effects of exogenous gene transfer, and/or the use of pharmacological reagents on retinal development [1-4]. Several advantages are afforded by the *ex-vivo* approach, including the ability to target a relatively synchronous population of retinal progenitor cells (RPC) for plasmid transfection via electroporation. Multiple aspects of retinal development, including detailed timing of interkinetic nuclear migration, terminal mitosis, radial migration, morphological and physiological maturity and connectivity can all be evaluated using this approach [1-10]. Central to this technique, is the ability to transfect cells with plasmid DNA constructs driven by either ubiquitous or cell type-specific promoters [1-3], as well as gain-of-function and loss-of-function vectors. Fluorescent reporter genes located within knockdown vector constructs or co-transfected alongside knockdown plasmids permit the identification and morphological assessment of transfected cells in time and space.

A number of publications have highlighted the advantages afforded by live imaging during retinal development [5,7-9,11]. With the use of the transparent zebrafish embryo, movement kinetics of RPCs, cell specification, and terminal mitoses can be imaged, tracked and interrogated with the aid of transgenic approaches [5,11]. Although the rodent retina does not exhibit the same degree of optical accessibility and transparency to that of zebrafish embryos, imaging of explanted rodent retinas has been reported [6,12], and has provided valuable data pertaining to retinal cellular movement and physiology. The use of higher penetrating power, 2-photon microscopy has also expanded our ability to view deep tissue phenomena. A protocol describing the use of an adaptable and portable imaging system for use with 2-photon analysis would greatly improve our ability to temporally monitor rodent retinal development.

In this report, we describe a method of maintaining explanted mouse retinas in a culture preparation that is accessible to a wide assortment of inverted and upright imaging systems. We have modified our protocols to employ a CO_2_-independent media, and a simple, portable imaging chamber system. Examples of live imaging data and analysis demonstrate that nuclear and cytoplasmic morphology, as well as detailed data pertaining to interkinetic nuclear migration can be characterized using fluorescent reporters. Analysis of detailed movement kinetics with the use of hierarchical clustering analytic algorithms revealed a complex heterogeneity in nuclear migration that is sensitive to gene disruption. Collectively, these methods provide a robust assay for evaluating movement kinetics, cell morphometry, and migration in the developing rodent retina, and provide new insight into the detailed kinetics of RPC function.

## Results

### CO_2_-independent, live retinal explant culture, immobilization and imaging

**Figure 1:**
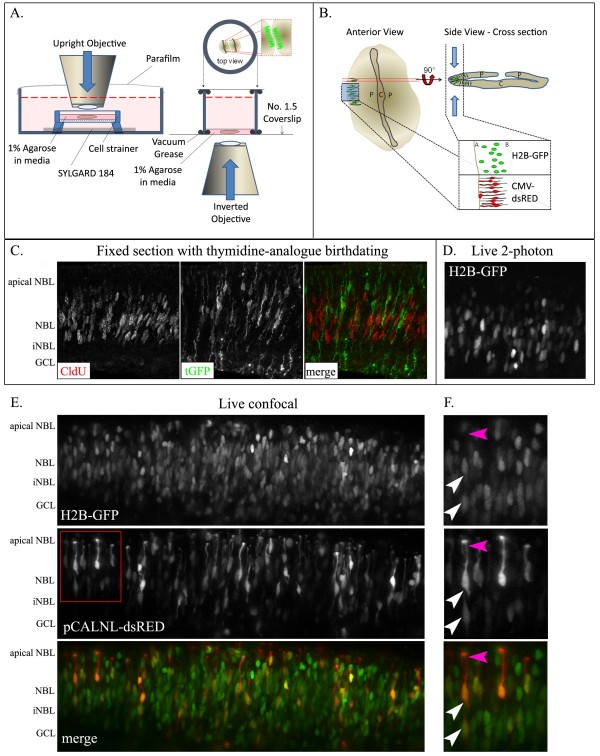
**Preparation and live imaging of postnatal mouse retinal explants.** (**A**) Schematic representations of upright and inverted imaging preparations. (**B**) Retinal and imaging orientations for use with cellular movement and morphology analysis. P = peripheral retina; C = central retina; A = apical neuroblastic margin; B = basal neuroblastic margin. (**C**) An example of conventional histological data in which thymidine analogue (CldU for 30 minutes at 29.5h post-electroporation) immunolabeling provides a means to evaluate the position and morphology of s-phase and non-s-phase retinal cells expressing HuSH-scrambled shRNA-tGFP. NBL = neuroblastic layer; GCL = ganglion cell layer. (**D**) An example of live 2-photon imaging in retinas transfected with H2B-GFP at P0, and imaged 20h later. (**E**) Live, 2-channel confocal imaging of retinas co-transfected at P0 with H2B-GFP (300 ng/μl), a trace amount (50 ng/μl) of CMV-Cre, and the Cre-sensitive reporter pCALNL-dsRed (500 ng/μl; final ratio of 6:1:10). Images were acquired 48h post-transfection. (**F**) High magnification insets (red box in **E**). White arrowheads indicate variability in terminal nuclear position; magenta arrowheads indicate morphological features of cytoplasmic dsRed localization in apical processes.

To determine whether orthogonal assessment of RPCs can be evaluated in living mouse retinas, we developed a protocol for extended maintenance of immobilized explants for use with laser scanning microscopy (see Methods for a detailed protocol). P0 mouse retinas were dissected and transfected with a panel of fluorophore expression plasmids (Table 1) via square wave electroporation as previously described [1-4], with some modifications. In cursory evaluations under wide-field epifluorescence, we observed cytomegalovirus (CMV) promoter driven histone-2-B (H2B) - green fluorescent protein (GFP) fusion protein reporter (*CMV:H2B-GFP*) signal as early as ~15h post-electroporation (not shown). Following 20h of culturing on polycarbonate membranes, retinas were transferred to a custom-assembled CO_2_-independent imaging chamber adapted for imaging on either an inverted or upright microscope (Figure 1A). Following immobilization with agarose-supplemented media, retinas were maintained in normal atmospheric conditions at 37 degrees C for up to 7-days. Fixed tissue histological comparison of agarose-embedded explants with age matched (P3) *in vivo* and non-embedded explant controls revealed a high preservation of cell distribution, composition, and overall retinal integrity when cultured in agarose for 48h (Additional file 1: Figure S1). Despite the presence of GFP-transfected cells in explants cultured in agarose for 7 days, histological evaluation revealed a loss in retinal adhesion and the presence of rosette formation at this time (not shown). Based on these observations, modifications to this protocol would need to be employed in order to organotypically culture beyond 48h.

**Table 1:** DNA constructs used in this study

**Plasmid**	**Source**	**Localization**	**Reference**
HuSH-scrambled shRNA-tGFP	Origene	Nuclear and cytoplasmic	-
CMV:H2B-GFP fusion	P. Howard	Nuclear	Manuscript in preparation
CMV:Ars2-GFP fusion	P. Howard	Nuclear	Manuscript in preparation
CMV-Cre	Addgene	Nuclear	Matsuda & Cepko, *PNAS*, 2007
CALNL-DsRed	Addgene	Nuclear and cytoplasmic	Matsuda & Cepko, *PNAS*, 2007
NRL-DsRed	Addgene	Nuclear and cytoplasmic	Matsuda & Cepko, *PNAS*, 2007

With the use of laser-scanning confocal microscopy, agarose-embedded retinas were live imaged (Figure 1D-F), and were compared to temporally matched (29.5h) samples of fixed retinal sections that had been maintained on polycarbonate membranes, and that were prepared using conventional histological techniques (Figure 1C). Evaluation of both cytoplasmic and nuclear-localized fluorescent labeling in all explants tested revealed no discernible difference in retinal architecture when comparing live and fixed samples. Similar to a previous study [12], live imaging at the edge of explants revealed a folded portion of the retina that provided a cross-sectional view across the apical-basal axis (inset schematic in Figure 1B). The advantage of this orientation is that most of the cellular movements (i.e. interkinetic nuclear migration) occur along the apical/basal axis, parallel to the x-y plane. Thus, with the apical edge of the retina serving as a visual landmark it is much easier to correct z-axis projections for growth-related changes in cell position.

**Figure 2:**
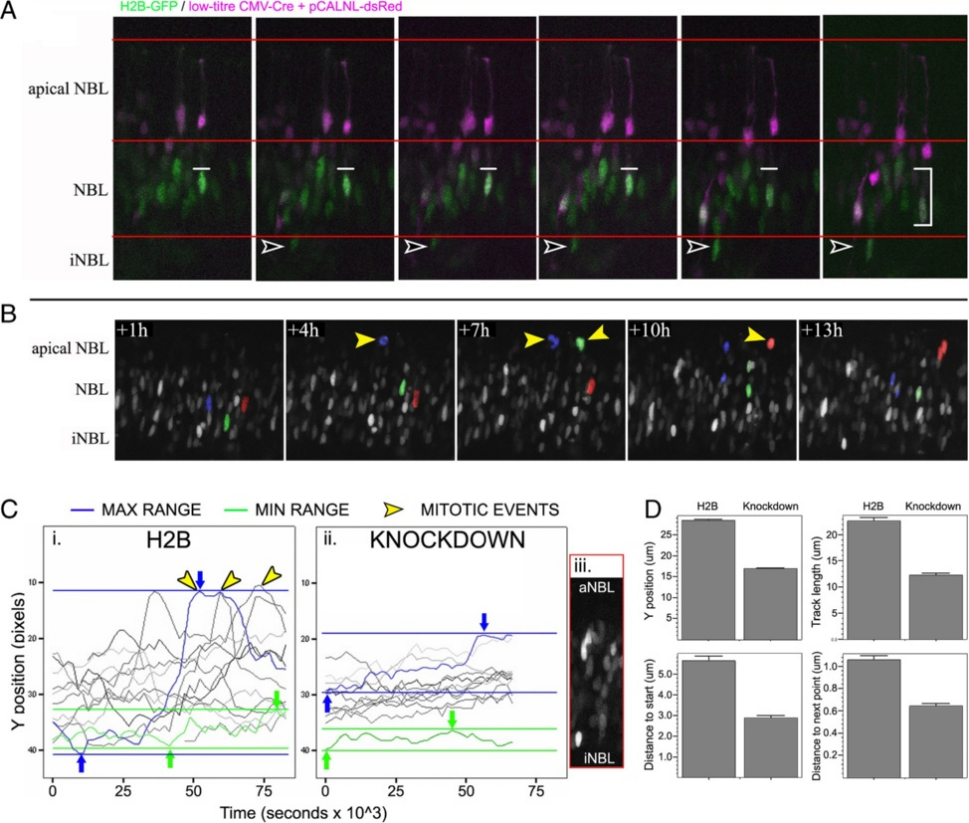
**Visualizing movement kinetics in the postnatal mouse retina.** (**A**) A time series demonstrating changes in morphology and position of transfected retinal cells. Postnatal day 0 mouse retinas were co-electroporated with a nuclear-localized GFP, CMV-Cre and pCALNL-dsRed. Image acquisition began at 24h post-transfection with a 3h per frame temporal resolution. Open arrowheads indicate an emerging eGFP nucleus in the differentiating iNBL. Bracket in the final frame indicates the range of movement by a nucleus within a GFP/dsRed co-localized cell. NBL – neuroblastic layer; iNBL – inner neuroblastic layer. (**B**) A selection of images from a time series demonstrating mitotic events (yellow arrowheads) by H2B-GFP expressing nuclei. (**C**). Cell tracking histograms of nuclear movements for H2B-GFP (i.) and knockdown (ii.) retinas. Blue lines and arrows represent the largest range of movement by a single nucleus, whereas green lines and arrows represent the smallest range. Yellow arrowheads indicate mitotic events, and correspond to yellow arrowheads in (**B**). (iii) is a representative live frame of H2B-GFP distribution across the apical NBL margin (aNBL) and iNBL. (**D**) Comparison of H2B-GFP control nuclear tracing data with knockdown nuclei.

To test whether detailed morphological assessment of individual RPCs could be viewed in tandem with nuclear positioning, *CMV:H2B-GFP* plasmids were co-electroporated with *CMV:Cre* and a conditional DsRed reporter plasmid, *pCALNL-DsRed* that is turned on following cre-mediated removal of a floxed stop codon [3]. The *CMV:Cre* plasmid concentration was titrated down in an effort to generate unlabeled space between DsRed expressing cells, allowing for a detailed view of individual cells (Figure 1E-F). Two-channel confocal (Figure 1E-F) and 2-photon (Figure 2A) live imaging revealed expression of GFP and DsRed in all retinal explants tested.

**Figure 3:**
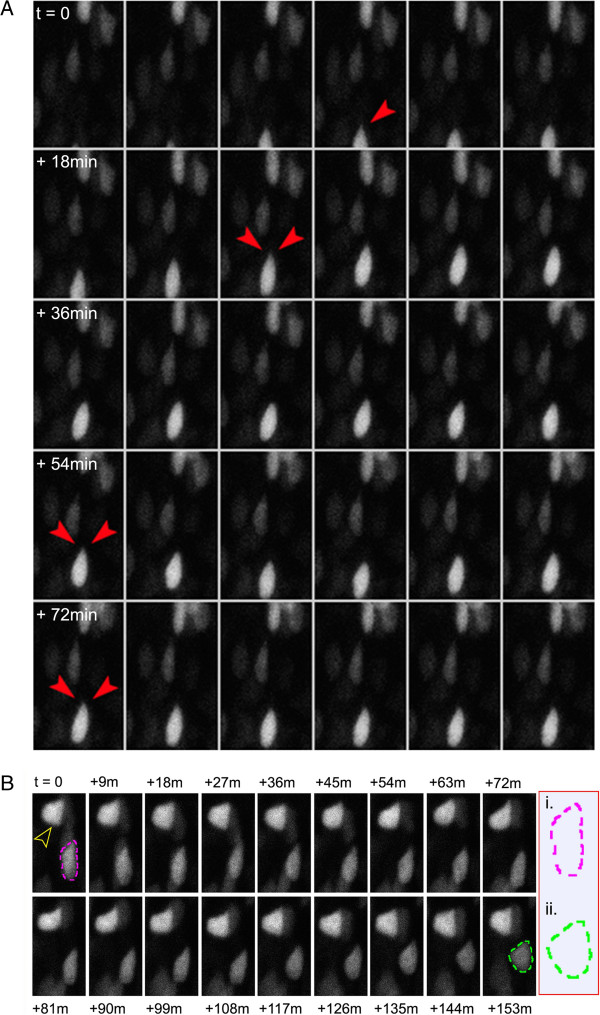
**Detailed changes in nuclear morphology during retinal development.** A high temporal resolution (3 minutes per frame), 2-photon image series demonstrating periodic changes in nuclear morphology during apical/basal movements. (**A**) A highly motile nucleus demonstrating advancing movement. Red arrowheads indicate the emergence of wisp-like protrusions in the otherwise smooth apex of an H2B-GFP expressing nucleus. (**B**) Immotile nuclei imaged over a 153 minute time course. Yellow arrowhead indicates a relatively static nuclear morphology exhibited by what appears to be a differentiated cell. Outline (inset in i. and ii.) depicts a rapid morphological shift from progenitor-like, to a more differentiated nuclear appearance.

To acquire a detailed view of morphological changes exhibited by transfected nuclei over time, we used 2-photon microscopy to acquire time series z-stacks using a 3-minute scanning interval starting at approximately 20h post-electroporation (Figure 3A-B, Additional file 2). At this level of temporal resolution, a detailed view of nuclear movement was evident, which allowed for the identification of both motile and immotile RPC nuclei. We observed clear changes in nuclear morphology in highly motile progenitors, including apical nuclear protrusions during apical migration (Figure 3A, arrowheads). Furthermore, evaluation of stationary cells revealed what appeared to be a maturing morphological transition from an elongated to a more rounded nucleus (Figure 3B).

### Cell tracking

To determine whether a protracted view of movement kinetics can be evaluated in live mouse retinas, we acquired z-stacks using 2-photon microscopy at 30 minute to 1 hour intervals for durations up to 48h. Time lapse movies demonstrated H2B-GFP labeled nuclei display robust and heterogeneous patterns of interkinetic nuclear migration and terminal mitosis during early postnatal development (Figure 2A, B, Additional file 3: Figure S2, Additional files 4, 5, 6). To generate quantitative data pertaining to cellular movement, we tracked the position of individual nuclei over time using the MTrackJ plugin for ImageJ (see Methods). Data rendered as individual traces revealed a complex and highly heterogeneous population of RPC interkinetic nuclear movements (Figure 2C). To validate the utility of this approach to measure cell cycle kinetics and gene disruption, we compared H2B-GFP (control) nuclear traces to those nuclei in which cell cycle progression was disrupted using an Arsenate resistance protein-2-GFP fusion protein expression plasmid (Nickerson et al., manuscript in preparation). Ars2 has been shown to interfere with cell cycle progression in highly proliferative populations in plants and animals, and misexpression functions as a dominant-negative [13-17]. Evaluation of Ars2-GFP transfected retinas revealed a clear difference in Y-axis position over time, with reduction in both the minimum and maximum apical/basal ranges travelled by those nuclei (Figure 2C-D, Additional file 7).

### Statistical screening of RPC movement kinetics

**Figure 4:**
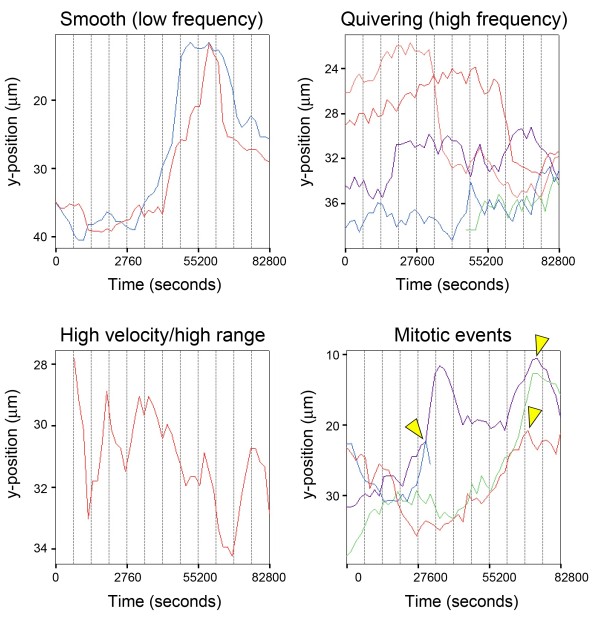
**Cell tracking.** Representative INM traces of nuclei belonging to one of four categories of movement (Smooth, Quivering, Erratic, Mitotic) identified through visual screening. Yellow arrowheads represent mitotic events.

**Figure 5:**
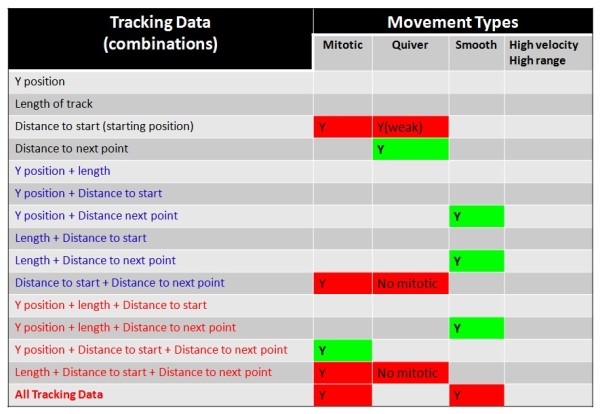
**Identification of hierarchical cluster screening algorithms.** Four types of movement data derived through cell tracking were used either independently (black text) or in 2 variable (blue) or 3 variable (red) combinations to hierarchically screen for nuclei that belong to one of the four categories of movement. Boxes containing a “Y” generate efficient clusters for that movement category. Green boxes represent exclusive clustering of cases belonging to that movement. Red boxes indicate clustering of cases from 2 or more movement categories, and were therefore, not useful for screening for individual movements.

**Figure 6:**
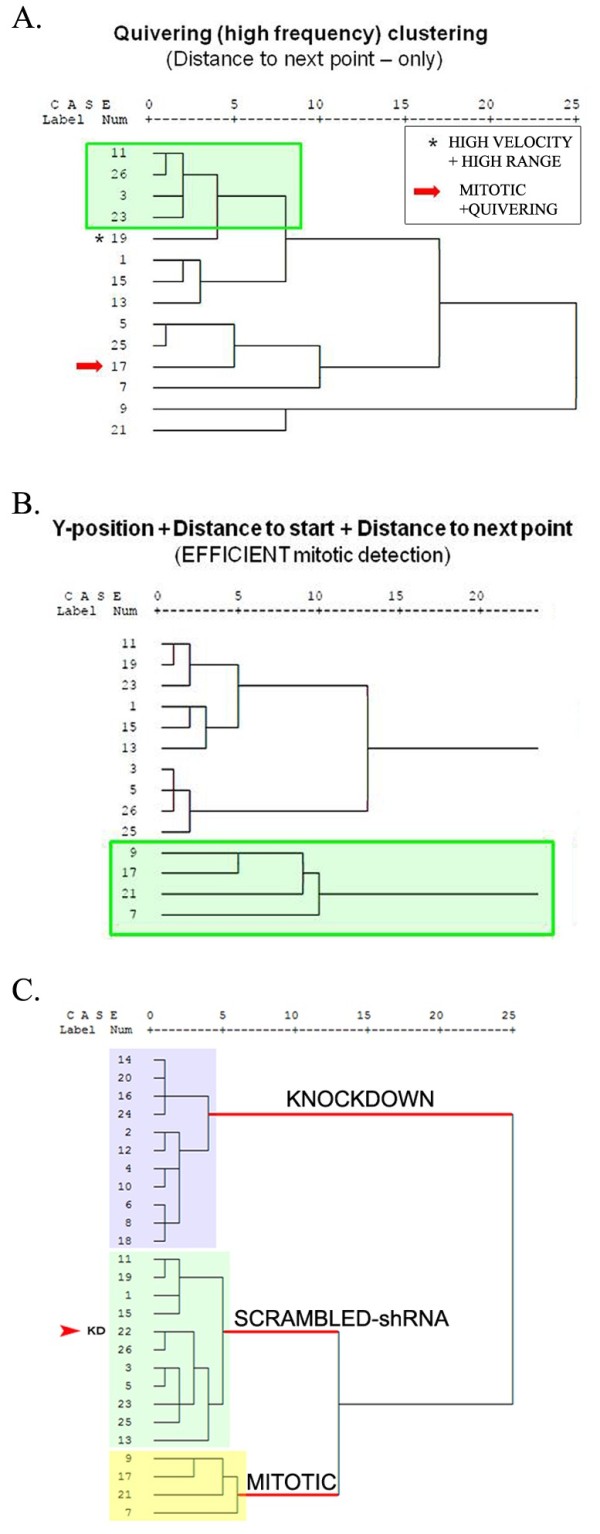
**Additional dependent measures of RPC movement kinetics correspond with increased clustering efficiencies.** (**A**) Example of a dendrogram output generated using *Distance to next point* as a dependent clustering variable, for the identification of cases that exhibit a quivering movement. Longer horizontal stems and fewer higher order branching points on the dendrogram output represent stronger clustering. Asterisk indicates contamination by a case from another movement category. Red arrow indicates a quivering nucleus that underwent a mitotic event, and was therefore clustered separately. (**B**) Dendrogram output demonstrating efficient clustering of mitotic nuclei (green box) using 3 dependent clustering variables. (**C**) Evaluation of changes in INM phenotypes using hierarchical clustering. Individual nuclei from control and knockdown groups can be identified and clustered with high accuracy (control = 100%; knockdown = 96%) from a merged data set using all four dependent variable measures. The red arrowhead indicates an erroneous assignment of a knockdown case to the control cluster.

In an effort to characterize the movement kinetics of tracked nuclei, we initiated a series of statistical screens with the aim of identifying specific analytic algorithms that can be used to isolate individual categories of RPC movements (see Hierarchical Screening). As a starting point, we identified four visually predominant categories of nuclear movement by reviewing individual raw data traces acquired for H2B-GFP labeled (i.e. control) nuclei (Figure 4). These categories include: 1) *Quivering nuclei*, which displayed a heterogeneous apical/basal range of movement, with a relatively high frequency of direction change; 2) *Smooth tracking nuclei*, which displayed a heterogeneous apical/basal range, but with a relatively low frequency of direction change; 3) *Erratic tracking*, which is comprised of a relatively homogeneous apical/basal range, with a relatively high frequency of direction change; and 4) *Mitotic events*. Lists of cases (individual nuclear traces) that exhibited one of the four identified categories of movement were compiled and used as a reference population for follow-up statistical screening. Our statistical screening method involved the use of hierarchical cluster analysis, based on the hypothesis that we would be able to accurately and reproducibly re-capitulate the membership of individual nuclear traces to the same categories of movement established though visual identification. The hierarchical cluster analytic approach was chosen, mainly because it is a simple and well-established statistical approach to segmentation and partitioning of multidimensional data in to groups. In our approach, clustering by this method is achieved using the Ward’s Method with Squared Euclidian Distances of hierarchical clustering, which calculates the statistical distance of individual data points, based on any one, all, or combination of ImageJ-generated dependent variables (summarized in Figure 5). Clusters are generated by minimizing the total within-cluster variance after merging cases. Data was first standardized into z-scores for use with this approach. As outlined in Figure 5, we used data (represented in pixels) acquired for (i) Y-position, (ii) length of each trace, (iii) distance to start, and (iv) distance to next point, for use with hierarchical clustering. Individual dependent variable data categories, as well as iterations of all combinations of those variables were used to comprehensively screen all clustering possibilities afforded by the tracking data. The resulting dendrogram output (examples in Figure 6A-B, Additional file 8) summarized the clustering products generated from each analysis. Dendrogram clusters that exhibit longer horizontal axis stems are defined as having stronger clustering relationships compared to those with shorter stems. Data were then screened for enrichment of cases that had been previously identified as belonging to individual movement categories. Our criteria for selecting algorithms for the identification of particular movement kinetics included that each algorithm be able to exclusively identify cases that belong to that movement category. By extension, we select against algorithms which identified a particular movement, but that also contained cases that belong to other movement categories. The results of our hierarchical cluster screen (summarized in Figure 5) indicated that the identification of individual movement kinetics can be isolated with high accuracy using either individual measures, or combinations of dependent variable data using this approach. Algorithms that included single, two and three dependent measures were all represented in accurate clustering solutions, indicating a need for multiple dependent measures for use in analyzing complex movement kinetics (Additional files 9, 10, 11).

To validate the utility of these screening algorithms, we analyzed tracking data acquired from 70 nuclei derived from 24 hours of live imaging (summarized in Additional file 12: Figure S3, Additional files 5 and 6). Using analytic algorithms identified from initial screening (above), we tested whether progressive inclusion of more dependent measures increased the efficacy of movement clustering (Additional file 9: Figure S4, Additional file 10: Figures S5, Additional file 11: Figure S6). Comparison of single variable (Distance to next point), bivariate (Y-position + Distance to next point) and multivariate (Y-position + Distance to next point + Distance to start + length) hierarchical clustering revealed an increase in the clustering efficiency contained within the solutions. For example, comparison of Distance to next point univariate clustering to that which uses all four measures revealed a striking increase in natural clustering, as defined by longer x-axis stems and visual inspection of traces. To test the applicability of this method in comparing control retinas to those in which the cell cycle was disrupted by expression of Ars2-GFP, we employed the same hierarchical screening method and compared these retinas to those transfected with H2B-GFP (Figure 6C). All tracking data from Ars2-GFP and H2B-GFP retinas were combined and randomized. Using all four dependent variable measures, our screen accurately clustered control nuclei with 100% accuracy, knockdown (KD) nuclei with 91.6% accuracy, and mitotic nuclei with 100% accuracy in merged and randomized datasets. Together, these data indicate that detailed movement kinetics can be isolated and compared statistically, both in the context of exploring movement characteristics, and when comparing control and experimental manipulations.

## Discussion

Live tissue imaging provides a means by which a relatively low number of explant preparations can be used to evaluate multiple time points, without affecting the statistical power required to evaluate the effect size. This is due, in part, to reduced variability when estimating treatment effects resulting from the processing of multiple samples, and increased power afforded by the repeated measures analysis. This approach is particularly advantageous during early pilot studies involving any characterization of effects over time, wherein 4D analysis with sufficient time resolution will allow one to precisely identify the onset of a treatment effect. High temporal resolution data pertaining to cellular movement, distribution and morphology are also highly informative, particularly with regard to interkinetic nuclear migration, mitosis, and terminal migration kinetics. When used in conjunction with cell-type-specific reporters, one can extend this analysis to include the onset of fate specification in relation to cell cycle exit.

### Live imaging and orientation

With the use of either confocal or 2-photon imaging systems, we can evaluate z-stacks acquired at regular intervals from a single live sample explant. This approach can be utilized to acquire time-series stacks in 4D, with the option of using single or multi-channel scanning acquisitions. Data from individual cells can be acquired through identification of transfected cells via fluorescent reporter constructs used in the transfection protocol (discussed below). One key aspect to this analysis is the orientation and visualization of the retinal cell layers, which becomes accessible during the natural folding of the retinal explant during procurement and culture (Figure 3) [6]. Careful microscopic exploration of the folded portion of the retina will reveal a “histologically-analagous” view of the retinal layering, which provides an anatomically familiar field to work with. This annulus of imageable tissue is comprised of retina located approximately half way between the central retina and peripheral margin (box in Figure 3). The use of cytoplasmic and nuclear reporter constructs (discussed below) provides an abundance of morphological reference for orientation, and can be used as reference points for any corrections in the xyz alignment over time. It is suggested that the experimenter adjust the amount of plasmid that is transfected according to the type of analysis being performed. For example, we have found that in nuclear tracking experiments, it is more difficult to distinguish individual cells in explants with a very high transfection efficiency.

### Plasmid selection

The selection of plasmids for use in these experiments is rapidly expanding, and includes anything from general fluorescent reporter expression, to overexpression constructs, knockdown vectors and physiological sensors. For gene disruption studies, we employ the use of fluorophore-expressing shRNA-based knockdown constructs (i.e. HuSH vectors, Origene). As an example, these vectors, which provide cytoplasmic and nuclear localized turbo-GFP, can be co-transfected with chromatin-binding (i.e. histone-2B) fluorescent fusion protein constructs. This approach, when using 2-channel scanning, provides the ability to discriminate between nuclear and cytoplasmic domains, and can be used to quantify phenotypes related to gene disruption. In addition to ubiquitous fluorophore expression, cell fate analysis can also be evaluated with the use of cell type-specific reporter vectors [1-3]. These reporter constructs contain promoter sequences that drive fluorophore expression during the specification of sub-types or classes of cells. Examples of commonly used retinal cell fate reporters include NRL- (photoreceptors), CRALBP (Müller glia), CaBp5 (subset of bipolars), and Hes1 (RPCs and Müller glia) [3], all of which are available through Addgene (http://www.addgene.org/). These plasmid-based reporter strategies can be used in conjunction with pharmacological manipulation, and the use of thymidine analogues for follow-up lineage tracing and cell cycle analysis. Upon completion of live imaging, explants can either be fixed for *post hoc* histological evaluation, or dissociated for use with flow cytometry.

## Conclusions

Our method offers a simple and affordable way to evaluate the kinetics of mammalian retinal development in live explants, and to analyze various features of RPC movement using a relatively simple series of statistical algorithms. Although this work focused on nuclear movement, our data also indicates that this method provides sufficient spatial and temporal resolution for use in many other areas of investigation, including terminal migration and evaluating subcellular changes in reporter localization.

## Methods

### Postnatal retinal explant dissection and in vitro electroporation

#### Dissection and electroporation

(Modified from Matsuda and Cepko, 2004, [modified from 3] and Donovan and Dyer 2006). See Table 2 for list of reagents and equipment. Note: All retinal dissection and electroporation steps should take place inside a sterile laminar-flow or microzone cabinet. Prepare counter space for decapitation and enucleation in close proximity to a sterile cabinet – include scissors, no. 5 forceps and 70% ETOH. The cabinet should contain a dissecting microscope and light source, two pairs of Dumont no.55 CO forceps, 1 pair of no.5 forceps, 1000 μl pipette with wide bore tips, 200 μl pipette with tips, 20 μl tips, prepared DNA solutions (see below), 100 μl electroporation chamber attached to electroporator, explant dissection dish containing pre-warmed HBSS without calcium or magnesium (-/-), media dish containing pre-warmed DMEM, explant wash dish containing pre-warmed HBSS(-/-). All experiments on mice were conducted with the approval of the University of Victoria Animal Care Committee.

**Table 2:** List of reagents and equipment

**Name of the reagent**	**Company**	**Catalogue number**	**Comments (optional)**
**Dissection and *in vitro *electroporation**			
Hank’s Balanced Salt Solution (-) Calcium Chloride, (-) Magnesium Chloride	GIBCO	14175	Denoted HBSS (-/-) in text
Hank’s Balanced Salt Solution (+) Calcium Chloride, (+) Magnesium Chloride	GIBCO	14025	Denoted HBSS (+/+) in text
Dulbecco’s Modified Eagle Medium (DMEM) high-glucose	GIBCO	11960	
Neurobasal Medium (-) glutamine	GIBCO	21103	
100 μl electroporation cuvette	Custom assembled		
ECM-830 square wave electroporator	BTX		
no. 55 Dumostar forceps	Fine Science Tools	11295-51	
no. 5 Dumont forceps	Fine Science Tools	11251-30	
Dissecting microscope	Nikon	SMZ-800	
Light source	Fiber-Lite	M1-150	
100x20 mm culture dish	Sarstedt	83.1802	
6-well culture dish	Starstedt	83.1839	
Polycarbonate Track-Etch Membranes	Whatman	110606	25mm, 0.2 um
B27 supplement	GIBCO	17504044	
Glutamax	GIBCO	35050-061	
1000 μl wide-bore pipette tips	Axygen	T-1005-WB-C-R	
200 μl pipette tips			Generic brand
20 up pipette tips			Generic brand
**Explant preparation for histology**			
HBSS (-/-)	GIBCO	14175	
4% paraformaldehyde in 0.1M PBS	Electron Microscopy Sciences	157-8	8% stock PFA
0.1M PBS			
0.1M PBS with 30% sucrose			
O.C.T. Compound	Tissue-Tek	4583	
Adhesive coated slides	Newcomer Supply	5070	75x25x1.0 mm

#### Explant culture dish preparation

Prepare 6-well culture dishes for culturing explants: 2.5 ml per well of explant media (Neurobasal media containing 1× Glutamax and 1× B27 supplement). Place a single polycarbonate membrane in each well with the shiny surface facing up. Place in incubator.

#### DNA preparation

Calculate the volume of DNA required to attain a final concentration rang of 0.1-1.5 μg/μl. For electroporation of 2–4 explants, prepare a total volume of 150 μl DNA in HBSS (+/+). For 5–8 explants, prepare 200 μl of DNA solution.

#### Explant preparation

Following decapitation of P1 mice outside of the biosafety cabinet, heads should be sprayed with 70% ethanol, eyes quickly enucleated, and placed into sterile HBSS(-/-). Retinas are dissected using two sterile Dumont no.5CO forceps. First, puncture the corneal-scleral interface with forceps, and progressively tease away the cornea. The retina can be removed from the eye cup, and the lens and ciliary epithelium/vitreous removed from the retina. Immediately transfer the retina to pre-warmed DMEM-high glucose or CO_2_-independent media using a wide bore pipette.

#### Electroporation

Rinse the retina in pre-warmed HBSS (-/-) and transfer to a 100 μl electroporation chamber. Remove HBSS and replace it with 100 μl of plasmid solution (1 μg/μl plasmid in HBSS(+/+). Using a no. 5 forceps that has been inserted into a 20 μl pipette tip to hold the tips closed, orient the retina such that the outer (scleral) surface faces the anode (or negative pole). Electroporate using the following parameters: Mode = Low-Voltage (LV), Set voltage to 30 Volts, Pulse length = 50 ms, Number of pulses = 5, Pulse interval = 950 ms. Remove DNA solution and return it to the original tube containing unused DNA (DNA ratio will be partially replenished using this method). Fill electroporation chamber with 100 μl HBSS(-/-). Transfer retina back to DMEM high-glucose. When all retinas (2–4 per plasmid) are complete, transfer explants onto polycarbonate membranes and cover with approximately 15 μl of explant media. Perform 50% media changes and re-apply 15 μl of media to each explant daily.

### Live imaging of retinal explants

#### Assembly of upright live imaging chambers

Begin by trimming a 50 ml Falcon-brand cell strainer (BD Falcon – Cat No. 352350) to a desired height for imaging with your upright microscope (Figure 1A). The strainer will be fixed into a 100 mm petri dish, so we suggest setting-up a mock assembly under your objective, and trim the strainer in a progressive fashion. The membrane height must be positioned sufficiently below the fill line for use with a dipping lens. 20-40× lenses are appropriate for use; we use a 20× lens (Olympus UMXXX, 2 mm working distance, 0.95 NA), which provides good resolution and a large field of view (sterilize the tip by wiping with 70% ETOH prior to immersing in culture medium). Fix the trimmed strainer to the bottom of a 100 mm petri dish using SYLGARD 184 (as per manufacturer’s instructions – Dow Corning Silicone Elastomer Kit). NOTE: When placing the strainer into the SYLGARD, be sure to leave sufficient space under the membrane for adequate contact with media below. Allow to set, rinse, and sterilize with 70% ETOH. Membranes can be cleaned with Tx-100, thoroughly rinsed, and sterilized in ETOH after each session of imaging.

#### Assembly of inverted live imaging chambers

Cover one edge of a sterile media chamber (we use either cloning rings or a small section of nylon tubing) with sterile vacuum grease and place it onto a no. 1.5 thickness coverslip (Figure 1B). We use shorter, 22 mm square coverslips, as added length adds to the fragility of the preparation. Test the seal by adding sterile HBSS to the chamber. Remove HBSS, and cover the upper edge of the media chamber with vacuum grease. Have a second coverslip ready to cap the media chamber following explant embedding (see below).

#### Preparing CO_2_-independent retinal culture medium

50 ml 1× CO_2_-independent culture medium (Gibco – cat. no. 18045, [-] Glutamine); 1× Glutamax; 1× B27 supplement, 50U of Pen Strep (Invitrogen – cat. no 15140–122).

#### Preparing 1% agarose in media

Add 0.01 g low melting temperature agarose (Gibco BRC) to 1 ml CO_2_-independent retinal culture medium. Prepare 2 samples (one for backup) and place in a heated block set at 70 degrees C. Monitor closely by visualizing the solution every 60 seconds while gently mixing. NOTE: Do not agitate. This will create small bubbles, which will significantly impair imaging. Once dissolved, allow agarose solution to cool while monitoring its temperature. Note: start the embedding protocol (below) when agarose solution reaches ~40 degrees C. Agarose will set quickly, so store in a 40 degree C block if necessary. We keep the second preparation at 70 degrees as a backup.

#### Embedding retinal explants

Transfected retinas are imaged as early as 15 hours following transfection. Prior to embedding, use wide field epifluorescence to identify regions of the explant that exhibit both sufficient fluorophore expression, and good retinal integrity. For imaging on an upright microscope, embed samples as follows (Figure 1A). First, place explant centrally on the membrane using a wide bore 1000 μl tip. Carefully remove excess media using a 200 μl tip, avoiding movement and damage to the explant. Gently add a drop of agarose solution over the explant and pause 5–10 seconds. Add additional agarose in a dropwise fashion until the explant preparation is totally immersed, and the agarose covers the extent of the membrane (agarose intercalates into the mesh stabilizing the explant and keeping the agarose from floating when medium is added). Allow to set for a minimum of 5 minutes. Be aware that the thickness of agarose cannot exceed the 2mm working distance of the dipping lens (2 mm for 20× 0.95 NA; 3 mm for 40× 0.8 NA). When filling the dish with pre-warmed retinal explant medium, start by dripping directly over the agarose. This will prevent the agarose from being lifted by the surface tension of the media when filling the chamber. Add media to submerge the explant about 3–4 mm below the surface. Parafilm should be collared over the objective lens and around the opening in the petri dish lid. This helps to maintain sterility and reduces fluid loss through evaporation. Typically about 0.5 ml of water will accumulate as droplets on the underside of the parafilm tent.

For imaging on an inverted microscope, place explant centrally within the chamber. Remove excess media, and embed with agarose as described above. Allow to set, add pre-warmed explant medium and cap the chamber with a coverslip.

#### Temperature control

Explants are maintained at 35–37 degrees C during imaging on a heated stage using a Tempcontrol 37–2 digital 2-channel temperature regulator (PeCon, Germany). Temperature is monitored and recorded by probe throughout the experiment using a Fluke 51 K/J thermometer fitted with a 1.5 mm diameter Teflon coated probe (Fluke Electronics, Canada) immersed in the media. Although the agarose embedded retinal preparation is very stable, xyz drift can occur during changes (> +/- 1 degree C) in media temperature, and for this reason, you should ensure that your final media temperature has stabilized (this takes 1–2 hours) before data acquisition. For both upright and inverted preparations, we set our heated stage to 42 degrees C, and continuously monitored the media temperature adjacent to the explant. With stable room temperature, we have found that automatic temperature feedback control is not necessary, given the thermal mass provided by the large chamber volume. When imaging for >36h, a 50% media change may be required. Removal and re-filling of media will cause a transient change in the position of the embedded explant relative to the lens if the temperature drops during the exchange. In our setup, the position of imaged cells returned to within a few micrometers as the temperature restabilized in approximately 20 minutes.

#### General imaging parameters using GFP and DsRed fluorophores

2-photon microscope: Images stacks were captured using a custom acquisition system based on the Igor (Wavemetrics) system platform which includes routines to permit automatic acquisition of z-stacks at predetermined intervals. Image acquisition was typically done using the following parameters: excitation wavelength = 890–900 nm; power at front of the objective = 18–28 mW, dwell time 1 μsec per pixel, resolution 0.15-0.2 microns per pixel, 1.5 micron step size, 3–6 frames averaged for each optical section, one image stack every 30 minutes. For studies solely examining cell migration, a resolution of 0.4 microns per pixel is sufficient as it significantly reduces file size. In general, we found that fluorescent protein signal could be detected at ~20 hours after transfection, and that this signal intensity increased over time and eventually stabilized by ~26 hours. Note that for the experiments shown here the large back aperture of the 20X lens was only half-filled which results in a lower effective NA and thus less efficient excitation. Fully filling the back aperture could be expected to reduce required power. 16-bit TIFF image files were exported to ImageJ and converted to 8-bit for processing. Confocal microscope: Low laser outputs (0.5-1.5%), 1 AU pinhole, 1000 × 600 resolution, 8-bit, 1h intervals, 50–100 μm z-range stacks, 2-4X averaging, 1.64 ms dwell time. We combine wavelengths into 1 channel for increased speed and reduced photodamage. Images were processed by applying a median filter with a 1 pixel radius to reduce pixel noise.

### Cell tracking

Cell tracking in 4D can pose a number of challenges, including decisions to work with either orthogonal stacks or stack projections over time, as well as confronting issues with signal changes, and drift in x, y or z dimensions that may occur during multi-image acquisition. Due to the variability in imaging systems and available software packages, we will only briefly describe our cell tracking methodology using ImageJ software, and focus on a selection of statistical approaches that can be used for this type of data.

We acquire stacks across the depth of the retina at constant intervals. These stacks are imported into ImageJ as multiimage TIFFstacks. We then prepare maximum intensity projections for each time interval. In cases where there are a large number of transfected cells, we prepare projections of sub-stacks encompassing approximately 30 um of z-plane, reducing the number of cells to be tracked in each file. The individual projections for each time point and depth are then combined into a stack from which the position of nuclei as a function of time can be obtained. NOTE: for accurate kinetic analysis using statistics, it is important that each image stack be acquired at time-points that are evenly spaced. Bits of cellular debris or non-motile cells can serve as fiducial points to correct for drift in the x or y direction. To correct for this drift, we use a plugin called MultiStackReg (TurboReg). This plugin will detect the most fiduciary point in the series, and will align any images to a reference image of your choice (typically the first image in the series).

Once registered, your image sequence can be analyzed for cell movements using the MTrackJ plugin. This software allows the researcher to mark individual cells, and plot their position in xy throughout the time series. The application of an intensity heat map filter (Additional files 5 and 6) can aid in the identification and resolution of individual nuclei within densely transfected groups of RPCs. Tracked cells can be managed by grouping nuclei into clusters or experimental groups as appropriate. ImageJ will then allow you to export the file in Excel format, which will include data pertaining to cell position, fluorescence intensity, distance travelled, velocity, and relative distances. Files can then be compiled, exported, and used for statistical analysis.

### Hierarchical screening

Import data to SPSS. The example (Additional file 13: Table S1 - below) demonstrates a short dataset in which 19 time points per nucleus were acquired at 30 minute intervals. Each nucleus will have a “track” associated with it (trackID), which is composed of 19 individual points (pointID), which correspond to a pre-defined time function (time_seconds). The y-position (pixels) for each point is used to identify position, and to calculate the derivations of: (1) line length to any point (length), (2) physical distance to the start position at any point (D2start); and (3) physical distance to the next point.

To begin, generate z-scores for use with Hierarchical Clustering.

SORT CASES BY trackid group.

CASESTOVARS

/ID=trackid group

/GROUPBY=VARIABLE.

DESCRIPTIVES VARIABLES=ypos_um.1

ypos_um.2 ypos_um.3 ypos_um.4

ypos_um.5 ypos_um.6 ypos_um.7

ypos_um.8 ypos_um.9 ypos_um.10

ypos_um.11 ypos_um.12 ypos_um.13

ypos_um.14 ypos_um.15 ypos_um.16

ypos_um.17 ypos_um.18 ypos_um.19

lengt_um.2 lengt_um.3 lengt_um.4

lengt_um.5 lengt_um.6 lengt_um.7

lengt_um.8 lengt_um.9 lengt_um.10

lengt_um.11 lengt_um.12 lengt_um.13

lengt_um.14 lengt_um.15

lengt_um.16 lengt_um.17 lengt_um.18

lengt_um.19 d2start.2 d2start.3 d2start.4

d2start.5 d2start.6 d2start.7

d2start.8 d2start.9 d2start.10 d2start.11

d2start.12 d2start.13 d2start.14 d2start.15

d2start.16 d2start.17

d2start.18 d2start.19 d2nxpoin.2 d2nxpoin.3

d2nxpoin.4 d2nxpoin.5 d2nxpoin.6

d2nxpoin.7 d2nxpoin.8

d2nxpoin.9 d2nxpoin.10 d2nxpoin.11

d2nxpoin.12 d2nxpoin.13 d2nxpoin.14

d2nxpoin.15 d2nxpoin.16

d2nxpoin.17 d2nxpoin.18 d2nxpoin.19

/SAVE

/STATISTICS=MEAN STDDEV MIN MAX.

The resulting data table will be transformed to accommodate Hierarchical Clustering (Additional file 14: Table S2), and will include new z-converted variables (Additional file 15: Table S3).

We next employ iterations of a combinatorial screening approach that uses our four dependent measures (y-position, length, distance to start, distance to next point) for Hierarchical Clustering (Ward’s Method for Squared Euclidian distances). Clustering algorithms will include single, bivariate, and multivariate approaches (summarized in Figure 5).

CLUSTER Zypos_um.1 Zypos_um.2

Zypos_um.3 Zypos_um.4 Zypos_um.5

Zypos_um.6 Zypos_um.7 Zypos_um.8

Zypos_um.9 Zypos_um.10

Zypos_um.11 Zypos_um.12 Zypos_um.13

Zypos_um.14 Zypos_um.15 Zypos_um.16

Zypos_um.17 Zypos_um.18

Zypos_um.19 Zlengt_um.2 Zlengt_um.3

Zlengt_um.4 Zlengt_um.5 Zlengt_um.6

Zlengt_um.7 Zlengt_um.8 Zlengt_um.9

Zlengt_um.10 Zlengt_um.11

Zlengt_um.12 Zlengt_um.13 Zlengt_um.14

Zlengt_um.15 Zlengt_um.16

Zlengt_um.17 Zlengt_um.18

Zlengt_um.19 Zd2start.2 Zd2start.3

Zd2start.4 Zd2start.5

Zd2start.6 Zd2start.7 Zd2start.8

Zd2start.9 Zd2start.10 Zd2start.11

Zd2start.12 Zd2start.13 Zd2start.14

Zd2start.15 Zd2start.16

Zd2start.17 Zd2start.18

Zd2start.19 Zd2nxpoin.2 Zd2nxpoin.3

Zd2nxpoin.4 Zd2nxpoin.5 Zd2nxpoin.6

Zd2nxpoin.7 Zd2nxpoin.8 Zd2nxpoin.9

Zd2nxpoin.10 Zd2nxpoin.11

Zd2nxpoin.12 Zd2nxpoin.13

Zd2nxpoin.14 Zd2nxpoin.15 Zd2nxpoin.16

Zd2nxpoin.17 Zd2nxpoin.18 Zd2nxpoin.19

/METHOD WARD

/MEASURE=SEUCLID

/PRINT SCHEDULE

/PLOT DENDROGRAM VICICLE.

CLUSTER Zypos_um.1 Zypos_um.2

Zypos_um.3 Zypos_um.4 Zypos_um.5

Zypos_um.6 Zypos_um.7

Zypos_um.8 Zypos_um.9 Zypos_um.10

Zypos_um.11 Zypos_um.12 Zypos_um.13

Zypos_um.14 Zypos_um.15 Zypos_um.16

Zypos_um.17 Zypos_um.18

Zypos_um.19

/METHOD WARD

/MEASURE=SEUCLID

/PRINT SCHEDULE

/PLOT DENDROGRAM VICICLE.

CLUSTER Zlengt_um.2 Zlengt_um.3

Zlengt_um.4 Zlengt_um.5 Zlengt_um.6

Zlengt_um.7 Zlengt_um.8 Zlengt_um.9

Zlengt_um.10 Zlengt_um.11

Zlengt_um.12 Zlengt_um.13

Zlengt_um.14 Zlengt_um.15

Zlengt_um.16 Zlengt_um.17

Zlengt_um.18 Zlengt_um.19

/METHOD WARD

/MEASURE=SEUCLID

/PRINT SCHEDULE

/PLOT DENDROGRAM VICICLE.

CLUSTER Zd2start.2 Zd2start.3

Zd2start.4 Zd2start.5 Zd2start.6

Zd2start.7 Zd2start.8 Zd2start.9

Zd2start.10 Zd2start.11

Zd2start.12 Zd2start.13 Zd2start.14

Zd2start.15 Zd2start.16 Zd2start.17

Zd2start.18 Zd2start.19

/METHOD WARD

/MEASURE=SEUCLID

/PRINT SCHEDULE

/PLOT DENDROGRAM VICICLE.

CLUSTER Zd2nxpoin.2 Zd2nxpoin.3

Zd2nxpoin.4 Zd2nxpoin.5 Zd2nxpoin.6

Zd2nxpoin.7 Zd2nxpoin.8 Zd2nxpoin.9

Zd2nxpoin.10 Zd2nxpoin.11

Zd2nxpoin.12 Zd2nxpoin.13

Zd2nxpoin.14 Zd2nxpoin.15

Zd2nxpoin.16 Zd2nxpoin.17

Zd2nxpoin.18 Zd2nxpoin.19

/METHOD WARD

/MEASURE=SEUCLID

/PRINT SCHEDULE

/PLOT DENDROGRAM VICICLE.

CLUSTER Zypos_um.1 Zypos_um.2

Zypos_um.3 Zypos_um.4 Zypos_um.5

Zypos_um.6 Zypos_um.7

Zypos_um.8 Zypos_um.9 Zypos_um.10

Zypos_um.11 Zypos_um.12 Zypos_um.13

Zypos_um.14 Zypos_um.15

Zypos_um.16 Zypos_um.17 Zypos_um.18

Zypos_um.19 Zlengt_um.2 Zlengt_um.3

Zlengt_um.4 Zlengt_um.5 Zlengt_um.6

Zlengt_um.7 Zlengt_um.8 Zlengt_um.9

Zlengt_um.10 Zlengt_um.11

Zlengt_um.12 Zlengt_um.13 Zlengt_um.14

Zlengt_um.15 Zlengt_um.16

Zlengt_um.17 Zlengt_um.18 Zlengt_um.19

/METHOD WARD

/MEASURE=SEUCLID

/PRINT SCHEDULE

/PLOT DENDROGRAM VICICLE.

CLUSTER Zypos_um.1 Zypos_um.2

Zypos_um.3 Zypos_um.4 Zypos_um.5

Zypos_um.6 Zypos_um.7

Zypos_um.8 Zypos_um.9 Zypos_um.10

Zypos_um.11 Zypos_um.12 Zypos_um.13

Zypos_um.14 Zypos_um.15

Zypos_um.16 Zypos_um.17 Zypos_um.18

Zypos_um.19 Zd2start.2 Zd2start.3

Zd2start.4 Zd2start.5 Zd2start.6

Zd2start.7 Zd2start.8 Zd2start.9 Zd2start.10

Zd2start.11 Zd2start.12 Zd2start.13

Zd2start.14 Zd2start.15 Zd2start.16

Zd2start.17 Zd2start.18 Zd2start.19

/METHOD WARD

/MEASURE=SEUCLID

/PRINT SCHEDULE

/PLOT DENDROGRAM VICICLE.

CLUSTER Zypos_um.1 Zypos_um.2

Zypos_um.3 Zypos_um.4 Zypos_um.5

Zypos_um.6 Zypos_um.7

Zypos_um.8 Zypos_um.9 Zypos_um.10

Zypos_um.11 Zypos_um.12 Zypos_um.13

Zypos_um.14 Zypos_um.15

Zypos_um.16 Zypos_um.17 Zypos_um.18

Zypos_um.19 Zd2nxpoin.2 Zd2nxpoin.3

Zd2nxpoin.4 Zd2nxpoin.5 Zd2nxpoin.6

Zd2nxpoin.7 Zd2nxpoin.8 Zd2nxpoin.9

Zd2nxpoin.10 Zd2nxpoin.11

Zd2nxpoin.12 Zd2nxpoin.13

Zd2nxpoin.14 Zd2nxpoin.15 Zd2nxpoin.16

Zd2nxpoin.17 Zd2nxpoin.18

Zd2nxpoin.19

/METHOD WARD

/MEASURE=SEUCLID

/PRINT SCHEDULE

/PLOT DENDROGRAM VICICLE.

CLUSTER Zlengt_um.2 Zlengt_um.3

Zlengt_um.4 Zlengt_um.5 Zlengt_um.6

Zlengt_um.7 Zlengt_um.8 Zlengt_um.9

Zlengt_um.10 Zlengt_um.11

Zlengt_um.12 Zlengt_um.13

Zlengt_um.14 Zlengt_um.15

Zlengt_um.16 Zlengt_um.17

Zlengt_um.18 Zlengt_um.19

/METHOD WARD

/MEASURE=SEUCLID

/PRINT SCHEDULE

/PLOT DENDROGRAM VICICLE.

CLUSTER Zlengt_um.2 Zlengt_um.3

Zlengt_um.4 Zlengt_um.5 Zlengt_um.6

Zlengt_um.7 Zlengt_um.8 Zlengt_um.9

Zlengt_um.10 Zlengt_um.11

Zlengt_um.12 Zlengt_um.13

Zlengt_um.14 Zlengt_um.15

Zlengt_um.16 Zlengt_um.17

Zlengt_um.18 Zlengt_um.19 Zd2start.2

Zd2start.3 Zd2start.4 Zd2start.5

Zd2start.6 Zd2start.7 Zd2start.8

Zd2start.9

Zd2start.10 Zd2start.11 Zd2start.12

Zd2start.13 Zd2start.14 Zd2start.15

Zd2start.16 Zd2start.17 Zd2start.18

Zd2start.19

/METHOD WARD

/MEASURE=SEUCLID

/PRINT SCHEDULE

/PLOT DENDROGRAM VICICLE.

CLUSTER Zlengt_um.2 Zlengt_um.3

Zlengt_um.4 Zlengt_um.5 Zlengt_um.6

Zlengt_um.7 Zlengt_um.8 Zlengt_um.9

Zlengt_um.10 Zlengt_um.11

Zlengt_um.12 Zlengt_um.13

Zlengt_um.14 Zlengt_um.15

Zlengt_um.16 Zlengt_um.17

Zlengt_um.18 Zlengt_um.19 Zd2nxpoin.2

Zd2nxpoin.3 Zd2nxpoin.4 Zd2nxpoin.5

Zd2nxpoin.6 Zd2nxpoin.7 Zd2nxpoin.8

Zd2nxpoin.9 Zd2nxpoin.10 Zd2nxpoin.11

Zd2nxpoin.12 Zd2nxpoin.13

Zd2nxpoin.14 Zd2nxpoin.15 Zd2nxpoin.16

Zd2nxpoin.17 Zd2nxpoin.18 Zd2nxpoin.19

/MET

## Competing interests

Financial competing interests: none

Non-financial competing interests: none

## Authors’ contributions

PEBN, RLC and PLH conceived and designed the research. PEBN, RLC, KMR and KRD performed transfections and live imaging. JDB assembled the IGOR custom 2 photon programming. PEBN and NFC performed the statistical screening. PEBN, KRD and RLC wrote the manuscript. All authors read and approved the final manuscript.

## Supplementary Material

Additional file 1: Figure S1 Histological comparison of agarose embedded retinas with age-matched in vivo and non-agarose controls. Immunolabeling of retinal sections. (A-D) Control retina fixed directly from a postnatal day 3 (P3) mouse pup. (E-H) Retinal explant cultured from day of birth (P0) for 72 hours on top of a polycarbonate membrane in neurobasal media under 5% CO_2_. (I-L) Edge region of retinal explant that was cultured first for 24 hours atop a polycarbonate membrane, and then embedded in low melt agarose in CO_2_-independent media and culture for an additional 48 hours. Retinas were immunolabeled for recoverin (A,E,I), Vsx2 (B,F,J) and nuclei were stained with Draq5 (C,G,K). The yellow dashed lines in (L) indicates a region at the edge of the explant where live 2-photon live would normally be performed. The white dashed line indicates the basal boundary of the neuroblastic layer. Abbreviations: NBL - neuroblastic layer, GCL - ganglion cell layer. Scale bar in (L) = 50 microns for all panels. Click here for file

Additional file 2 High temporal resolution live imaging: P0 mouse retinas were transfected with H2B-GFP, cultured for 20h, and imaged at 3 minute intervals using 2-photon microscopy. Click here for file

Additional file 3: Figure S2 Detection of mitotic events using live imaging. Time series panel of retinas transfected with H2B-GFP at P0, cultured for 28h, and imaged for 13.5h (z-stacks at 30 minute intervals) using 2-photon microscopy. Individual mitotic events (circles) are exhibited by multiple nuclei (pseudo colored). Click here for file

Additional file 4 Protracted (24h) live imaging: P0 mouse retinas were transfected with H2B-GFP, cultured for 20h, and imaged at 30 minute intervals using 2-photon microscopy. Mitotic nuclei (colored) are clearly detectable throughout the duration of imaging, and exhibit large apical/basal ranges of movement. Click here for file

Additional file 5 Raw data of 24h live retinal imaging: P0 mouse retinas were transfected with H2B-GFP, cultured for 20h, and imaged for 24h at 30 minute intervals using 2-photon microscopy. Click here for file

Additional file 6 Heat map enhanced, 24h live retinal imaging: P0 mouse retinas were transfected with H2B-GFP, cultured for 20h, and imaged for 24h at 30 minute intervals using 2-photon microscopy. A heat map filter was used to aid in singlet nuclear discrimination during tracking. Click here for file

Additional file 7 Ars2-GFP transfected retinas imaged with 2P for 48h: P0 mouse retinas were transfected with Ars2-GFP, cultured for 20h, and imaged for 48h at 30 minute intervals using 2-photon microscopy. Click here for file

Additional file 8 Resulting dendrogram output from hierarchical clustering. Click here for file

Additional file 9 Low level clustering efficiency with the use of a single dependent screening variables. Click here for file

Additional file 10: Figure S7 Intermediate clustering efficiency using bivariate clustering variables. (Y-position + Distance to next point)+ Distance to start + Length as dependent clustering variables. Click here for file

Additional file 11 The highest level of clustering efficiency was observed when using all four (Y-position, Distance to next point, Distance to start, Length) dependent variables. Click here for file

Additional file 12: Figure S3 Raw traces of 70 nuclei transfected with H2B-GFP. Individual nuclear movement summary of retinas transfected with H2B-GFP at P0, cultured for 20h, and imaged for 24h (z-stacks at 30 minute intervals) using 2-photon microscopy. Click here for file

Additional file 13: Table S1 Example of mTrackJ (ImageJ) tracking output – imported and managed in SP. Click here for file

Additional file 14: Table S2 A view of transformed data. Click here for file

Additional file 15: Table S3 New “Z” variables are produced. Click here for file
