# Supplementary figures and images for: Live imaging and analysis of postnatal mouse retinal development
Source: BMC Dev Biol. 2013 Jun 10;13:24. doi: 10.1186/1471-213X-13-24 (PMC3698203; doi:10.1186/1471-213X-13-24)

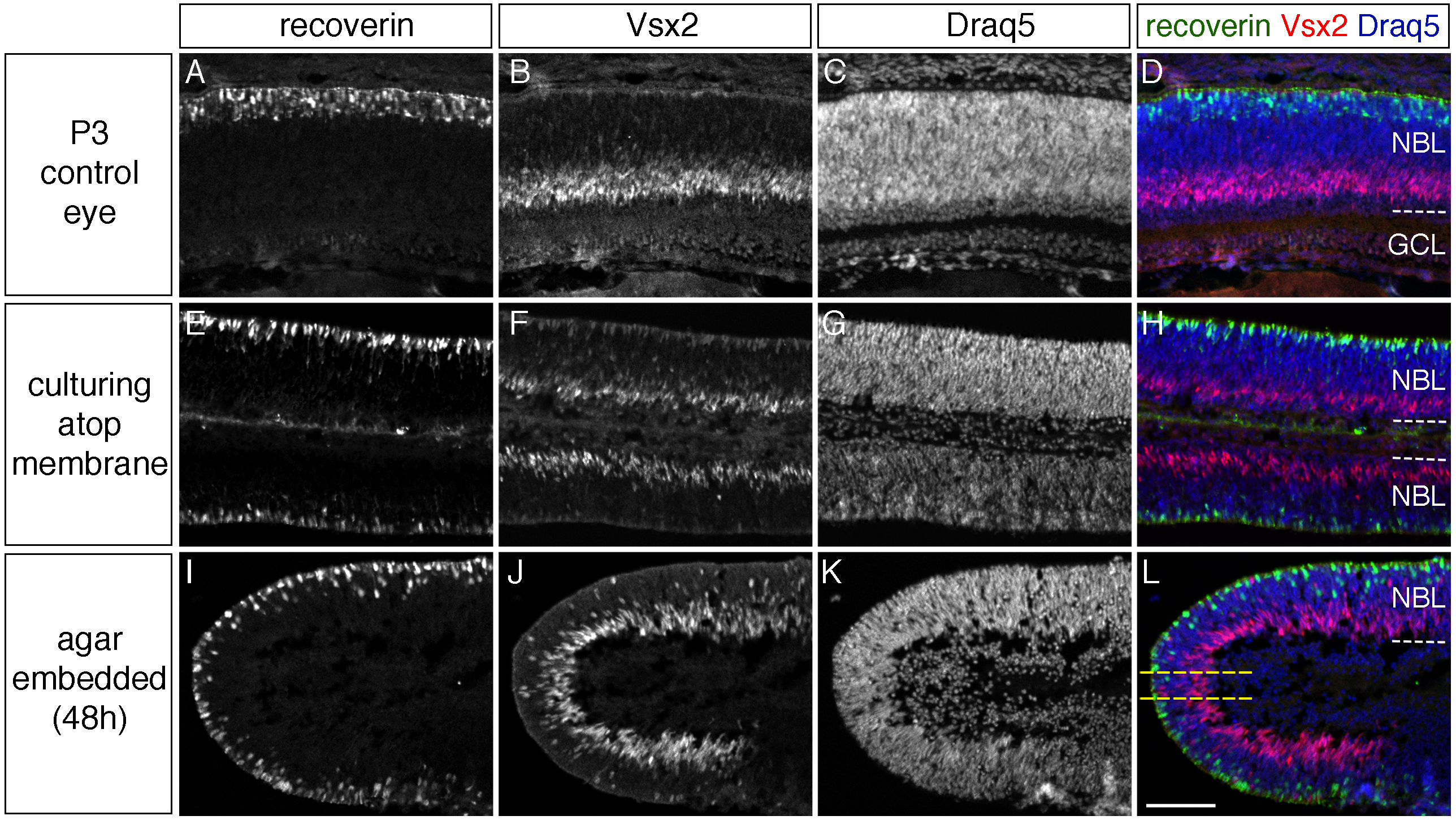

Supplement: Additional file 1: Figure S1 — Histological comparison of agarose embedded retinas with age-matched in vivo and non-agarose controls. Immunolabeling of retinal sections. (A-D) Control retina fixed directly from a postnatal day 3 (P3) mouse pup. (E-H) Retinal explant cultured from day of birth (P0) for 72 hours on top of a polycarbonate membrane in neurobasal media under 5% CO2. (I-L) Edge region of retinal explant that was cultured first for 24 hours atop a polycarbonate membrane, and then embedded in low melt agarose in CO2-independent media and culture for an additional 48 hours. Retinas were immunolabeled for recoverin (A,E,I), Vsx2 (B,F,J) and nuclei were stained with Draq5 (C,G,K). The yellow dashed lines in (L) indicates a region at the edge of the explant where live 2-photon live would normally be performed. The white dashed line indicates the basal boundary of the neuroblastic layer. Abbreviations: NBL - neuroblastic layer, GCL - ganglion cell layer. Scale bar in (L) = 50 microns for all panels. [file 1471-213X-13-24-S1.jpeg]

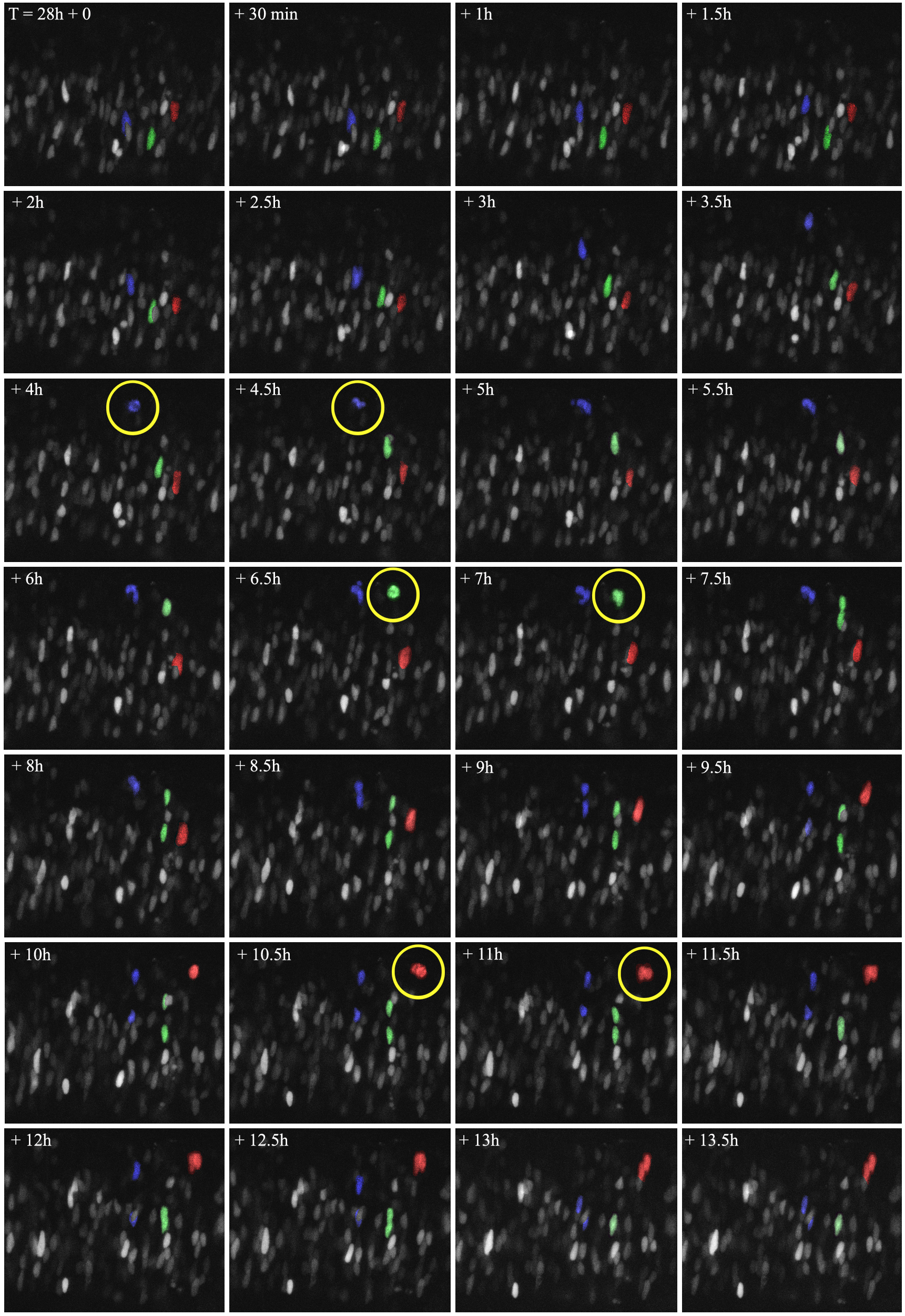

Supplement: Additional file 3: Figure S2 — Detection of mitotic events using live imaging. Time series panel of retinas transfected with H2B-GFP at P0, cultured for 28h, and imaged for 13.5h (z-stacks at 30 minute intervals) using 2-photon microscopy. Individual mitotic events (circles) are exhibited by multiple nuclei (pseudo colored). [file 1471-213X-13-24-S3.jpeg]

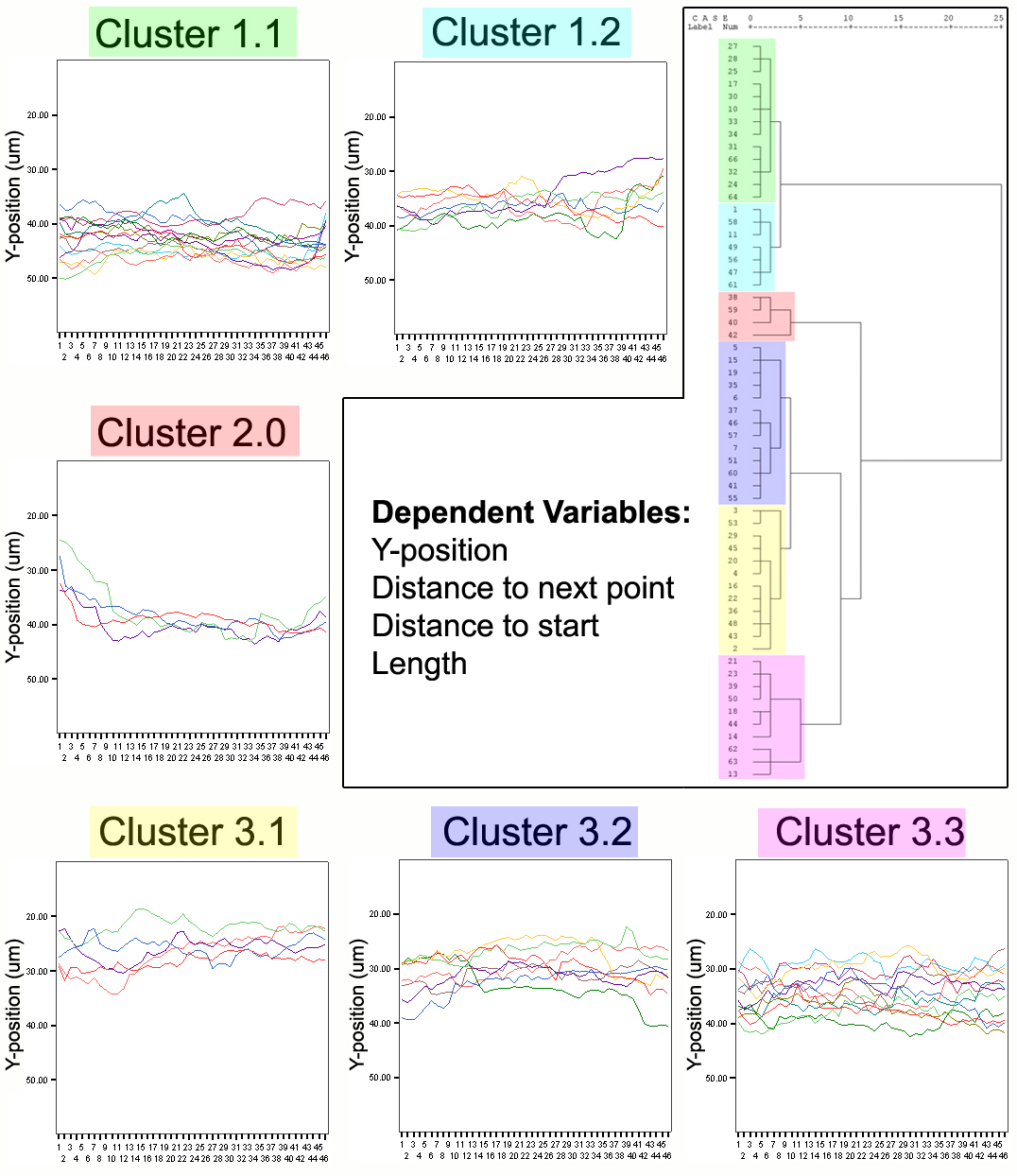

Supplement: Additional file 8 — Resulting dendrogram output from hierarchical clustering. [file 1471-213X-13-24-S8.jpeg]

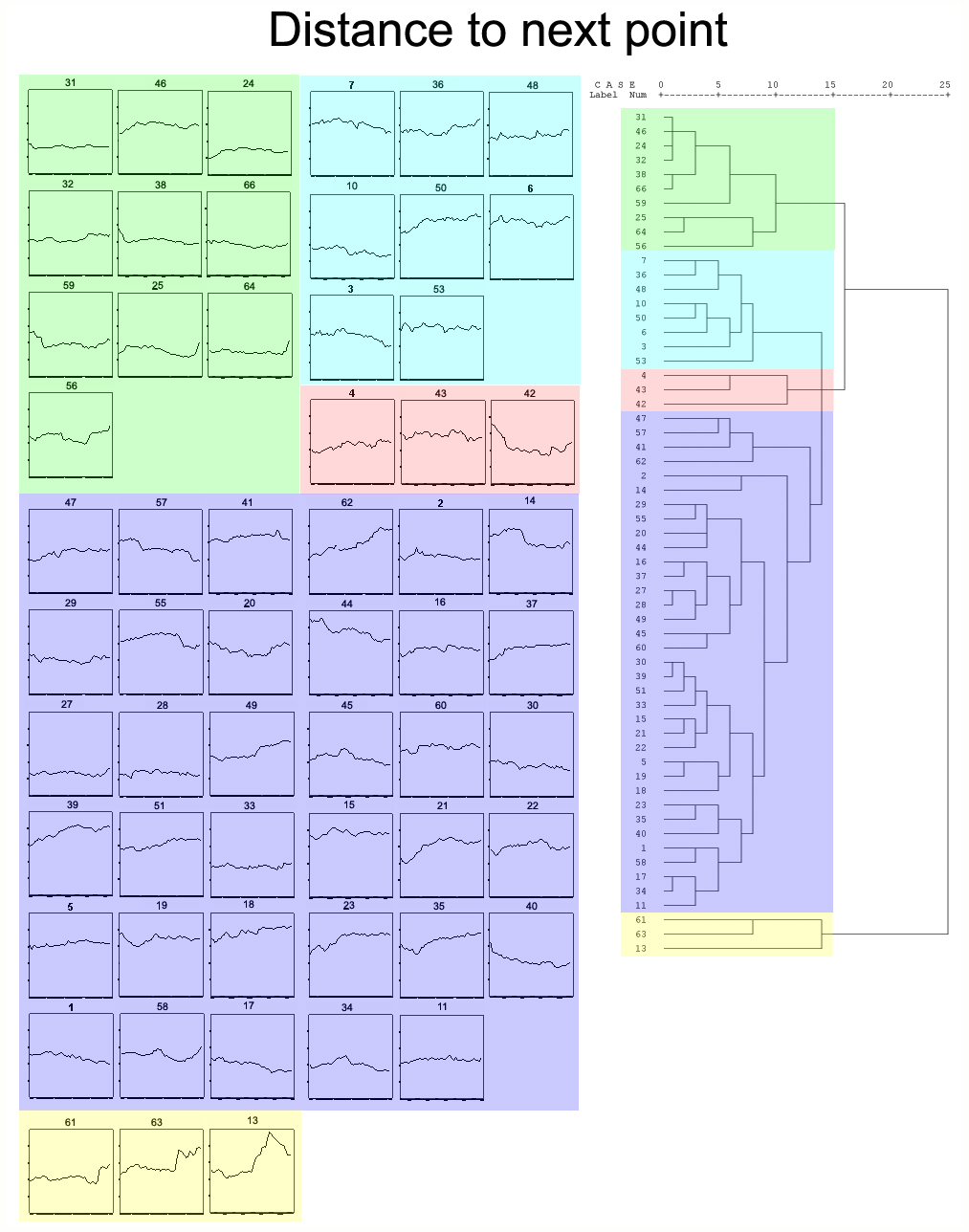

Supplement: Additional file 9 — Low level clustering efficiency with the use of a single dependent screening variables. [file 1471-213X-13-24-S9.jpeg]

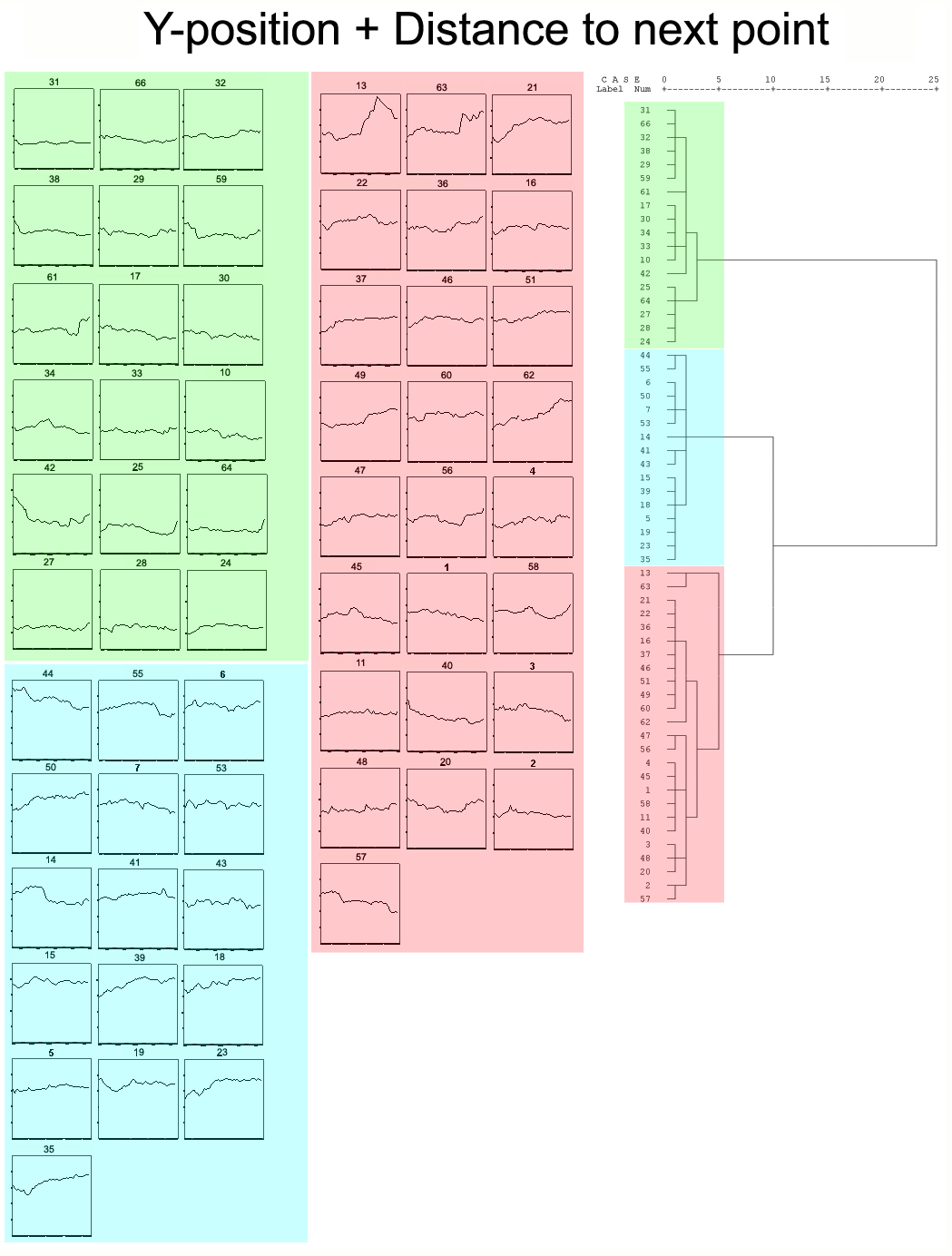

Supplement: Additional file 10: Figure S7 — Intermediate clustering efficiency using bivariate clustering variables. (Y-position + Distance to next point)+ Distance to start + Length as dependent clustering variables. [file 1471-213X-13-24-S10.jpeg]

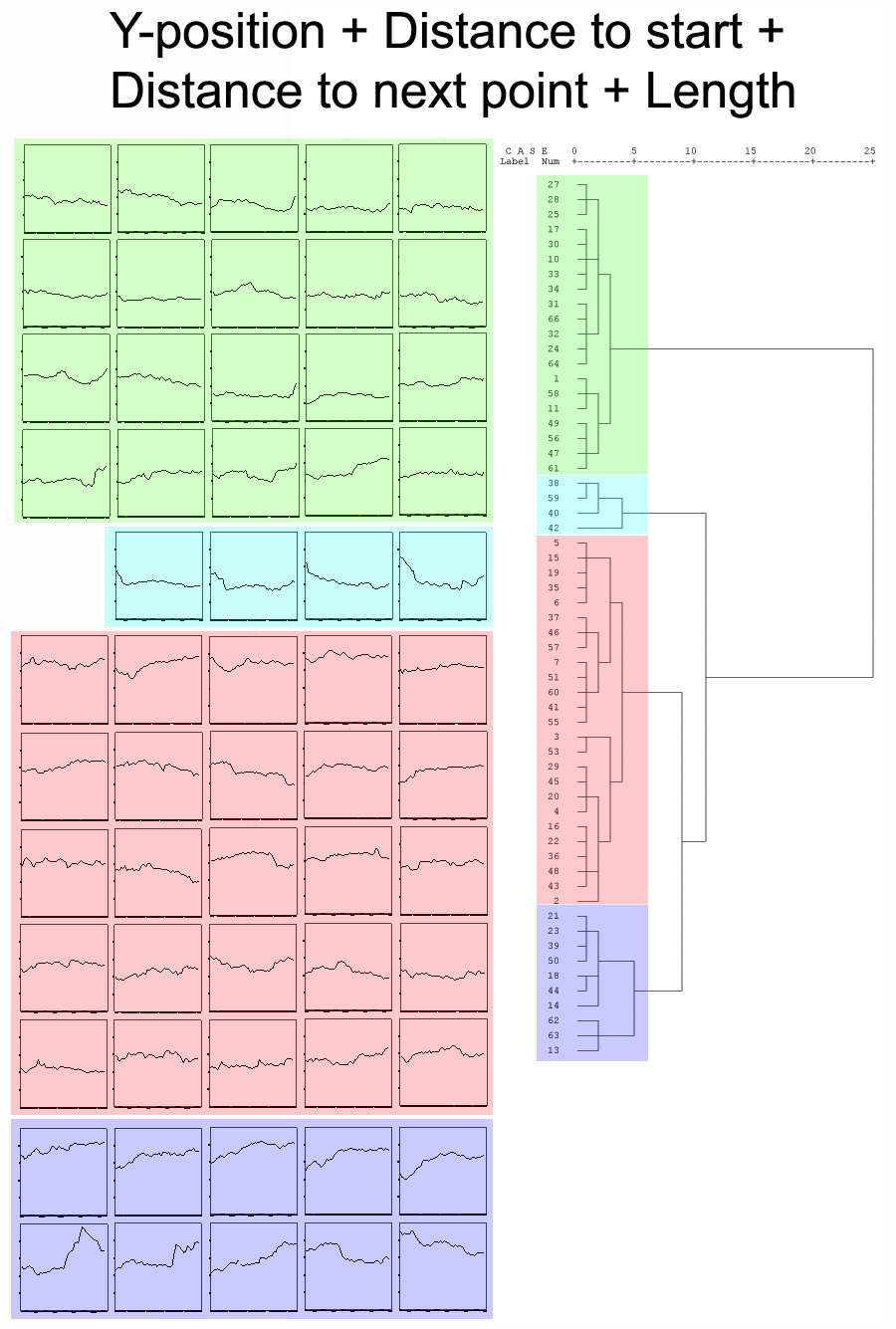

Supplement: Additional file 11 — The highest level of clustering efficiency was observed when using all four (Y-position, Distance to next point, Distance to start, Length) dependent variables. [file 1471-213X-13-24-S11.jpeg]

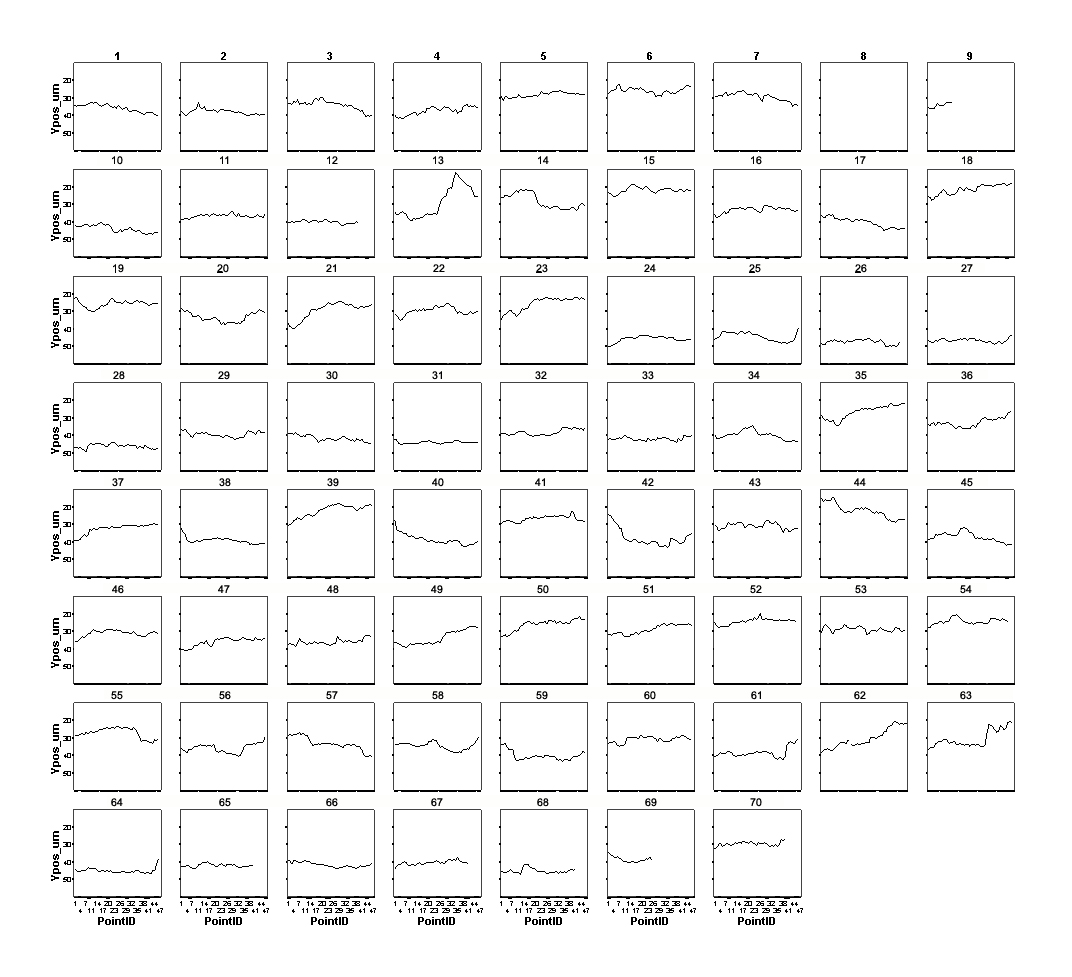

Supplement: Additional file 12: Figure S3 — Raw traces of 70 nuclei transfected with H2B-GFP. Individual nuclear movement summary of retinas transfected with H2B-GFP at P0, cultured for 20h, and imaged for 24h (z-stacks at 30 minute intervals) using 2-photon microscopy. [file 1471-213X-13-24-S12.jpeg]

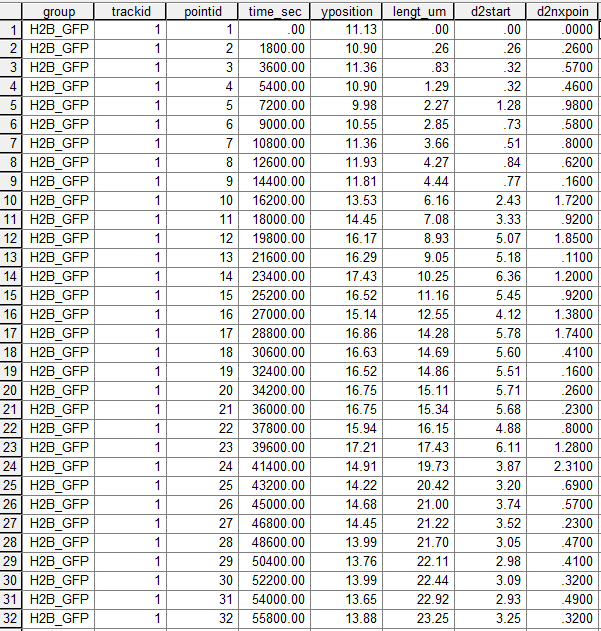

Supplement: Additional file 13: Table S1 — Example of mTrackJ (ImageJ) tracking output – imported and managed in SP. [file 1471-213X-13-24-S13.tiff]

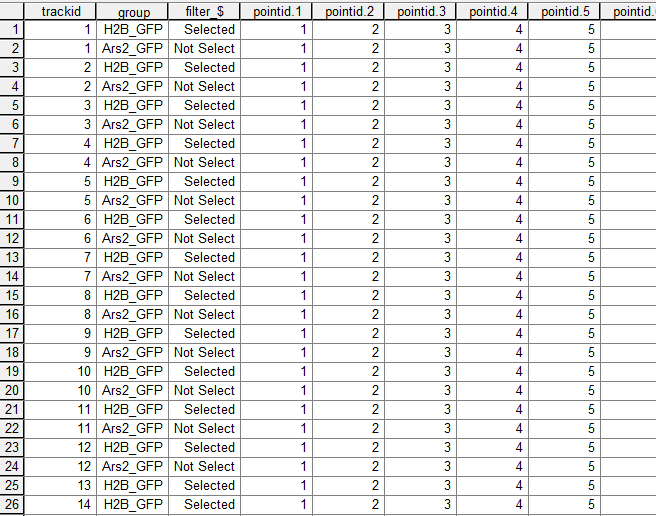

Supplement: Additional file 14: Table S2 — A view of transformed data. [file 1471-213X-13-24-S14.tiff]

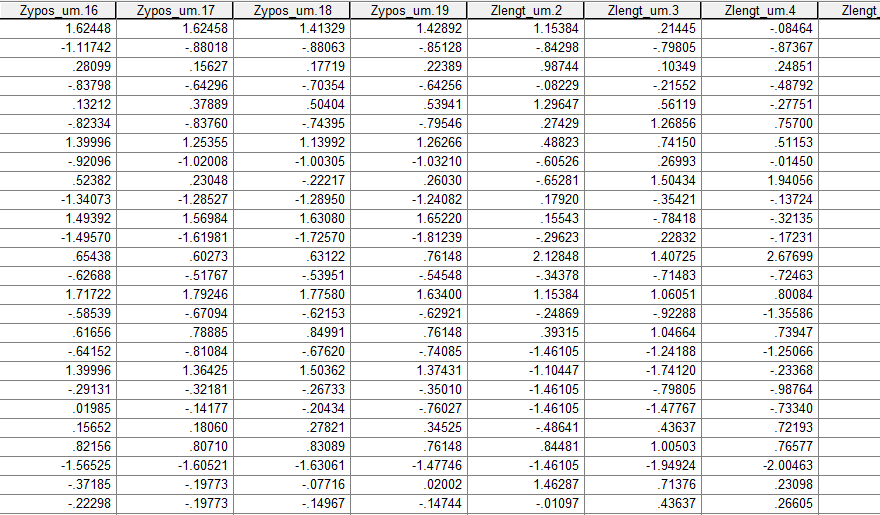

Supplement: Additional file 15: Table S3 — New “Z” variables are produced. [file 1471-213X-13-24-S15.tiff]
